# Examination of Jeltrate®Plus as a tissue equivalent bolus material

**DOI:** 10.1120/jacmp.v3i3.2560

**Published:** 2002-06-01

**Authors:** Steven Babic, Andrew T. Kerr, Mary Westerland, Jim Gooding, L. John Schreiner

**Affiliations:** ^1^ Kingston Regional Cancer Centre and Department of Oncology and Department of Physics Queen's University Kingston Ontario Canada K7L 5P9

**Keywords:** tissue equivalent bolus, radiation therapy, Jeltrate®Plus

## Abstract

A product available commercially for making dental impressions, Jeltrate®Plus, was evaluated as a tissue equivalent bolus material. Jeltrate®Plus was found to be tissue equivalent in 6 and 15 MV photon energy beams and 6, 12, and 20 MeV electron energy beams. As a first step, different preparations for making the bolus material were investigated and an optimal mixture was determined to be two parts Jeltrate®Plus powder to three parts water by weight. A suitable method for storing the material was found to be in a water filled plastic container. Since the product is fairly inexpensive and is easily and quickly made and moulded into different shapes, it is an excellent bolus material to use when treating irregular patient contours.

PACS number(s): 87.53.–j, 87.66.–a

## INTRODUCTION

In radiation therapy, bolus is often used when treating uneven surfaces of a patient such as around the nose or ears, to make up for missing tissue, or to provide build‐up and thus elevate the dose to the skin surface.^1^ ^,^ ^2^ In order to obtain the correct dose delivery, the material that makes up the bolus must have similar dosimetric properties as those of tissue. In addition to being tissue equivalent, the bolus material should be nontoxic, flexible enough to conform to surface contours, unaffected by high dose levels, easily made, cost effective, and durable.^3^ The purpose of this study was to assess the dosimetric properties and usefulness of a readily available dental mould, Jeltrate®Plus,^4^ as a bolus material.

## METHODS AND MATERIALS

**Figure 1:**
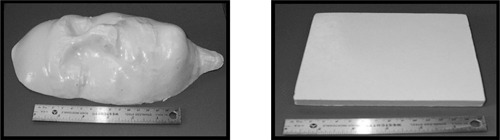
(a) Photographs of a facial mould to illustrate moldability and (b) a flat bolus slab made with a 2:3 Jeltrate®Plus powder: water mixture. The photograph on the left clearly shows the ability of Jeltrate®Plus to conform to irregular body contours. The ruler length is 15 cm.

A Braun®(Model MR 340) electrical household hand blender was used to mix Jeltrate®Plus (Dentsply Caulk, Milford, DE) powder and water at various concentrations. The concentration of powder to water was varied in ratios of 4:5, 2:3, 1:2, 1:3, and 1:4 by weight. Using a mould, a uniform $15\times 15{\text{cm}}^{2}$ bolus slab with a 1.3 cm thickness was created for each of the five different concentrations. A picture of a typical slab is shown in Fig. 1(b).

**Figure 2:**
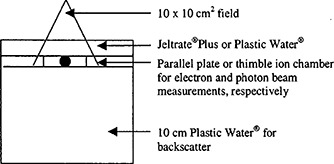
Phantom geometry for electron and photon beam ionization measurements. Electron and photon data were acquired with a 100 cm SSD and SAD setup, respectively. Either Jeltrate®Plus slabs or Plastic Water® was placed on top of the chambers depending on the experiment (see text).

Initial ionization measurements were made under each of the five slabs using an Attix parallel plate chamber (Radiation Measurements Inc., Middleton, WI). The radiation source was a 6 MeV electron beam from a Varian 2100 C/D linear accelerator. Data were acquired with each slab at a 100 cm SSD (refer to Fig. 2). Using the same set‐up, ionization measurements were performed under 1.3 cm of the water equivalent plastic, Plastic Water® (Nuclear Associates, Carle Place, NY),^5^ in order to obtain a percent ionization reading of the varying Jeltrate®Plus concentrations with respect to Plastic Water®. All of the bolus slabs were stored in separate water filled containers so as to prevent drying. Measurements were performed weekly for one month. Along with the quantitative measurements, the slabs of various powder to water ratios were compared to one another qualitatively in terms of their flexibility, durability, and length of production time. From the results of these comparisons, the 2:3 Jeltrate®Plus powder to water concentration was selected as the optimal bolus concentration. The subsequent measurements were all performed using bolus with this concentration.

A second set of measurements was made to investigate tissue equivalency of the 2:3 Jeltrate®Plus powder to water concentration by performing percent depth ionization measurements in water equivalent plastic using a 100 cm SSD setup and the Attix parallel plate chamber. Measurements were made at varying depths in Plastic Water® both with and without a 1.3 cm thick slab of Jeltrate®Plus on the surface. The field size was set to $10\times 10{\text{cm}}^{2}$. The measurements were made with 6, 12, and 20 MeV electron beams from a Varian 2100 C/D linear accelerator.

In order to verify tissue equivalence over the range of bolus thickness that might be utilized clinically, ionization measurements were then made under varying thickness of both Jeltrate®Plus and Plastic Water®. These measurements were made using 6, 12, and 20 MeV electron beams and 6 and 15 MV photon beams from a Varian 2100 C/D linear accelerator. The electron beam measurements were performed using the Attix parallel plate chamber and a 100 cm SSD setup. The photon beam measurements were performed using a Capintec PR‐06C/G thimble ion chamber (Capintec Inc., Ramsey, NJ) and a 100 cm SAD setup. For both electron and photon beam measurements, different thicknesses of 2:3 Jeltrate®Plus slabs or Plastic Water® were placed on top of the chamber block (see Fig. 2).

## RESULTS AND DISCUSSION

**Figure 3:**
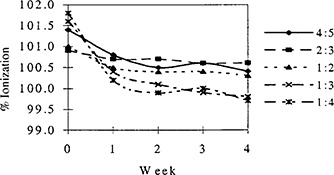
The values of ionization readings in Jeltrate®Plus relative to those in Plastic Water®, expressed as a percentage. The readings are for varying concentrations of Jeltrate®Plus powder in water (specified as the Jeltrate®Plus: water weight ratios in the legend). The data are for 6 MeV electrons.

The structural characteristics and tissue equivalency of Jeltrate®Plus bolus are expected to depend on the ratio of Jeltrate®Plus powder to water in the preparation. Figure 3 shows the results over one month of weekly ionization measurements made under bolus prepared with varying concentrations of Jeltrate®Plus powder in water. The duration of this stability evaluation was chosen as it corresponded to a typical patient treatment time. The initial percent ionization readings of freshly prepared bolus with varying Jeltrate®Plus concentrations were approximately 0.7–1.7% different than the readings under the water equivalent plastic, Plastic Water®. Measurements made with all concentrations of Jeltrate®Plus during the subsequent weeks were within 0.7% of Plastic Water® ionization measurements.

The results of the qualitative examination of the slabs can be found in Table I. They indicate that the 2:3 ratio Jeltrate®Plus powder to water meets all three of the desired bolus characteristics of being flexible, durable, and quick to make. As a result, this 2:3 Jeltrate®Plus powder to water concentration by weight was considered to be the optimal concentration for clinical use.

**Table I:** Physical characteristics of bolus made using various concentrations of Jeltrate®Plus powder in water.

Concentration by weight	Flexibility	Durability	Time for slabtoset
4 to 5	not flexible	very durable	<1min
2 to 3	flexible	very durable	1–2 min
1 to 2	flexible	durable	5 min
1 to 3	very flexible	fairly durable	5–10 min
1 to 4	very flexible	not durable	>10min

**Figure 4:**
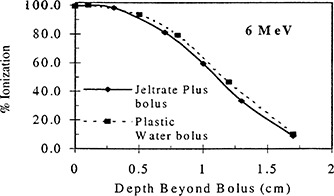
Central axis depth ionization curves for 6 MeV electrons measured beyond 1.3 cm of either Jeltrate®Plus bolus or Plastic Water® for a $10\times 10{\text{cm}}^{2}$ field and 100 cm SSD. The data for Jeltrate®Plus bolus are expressed as a percentage of the ionization at $d_{\max}$ in Plastic Water®.

**Figure 5:**
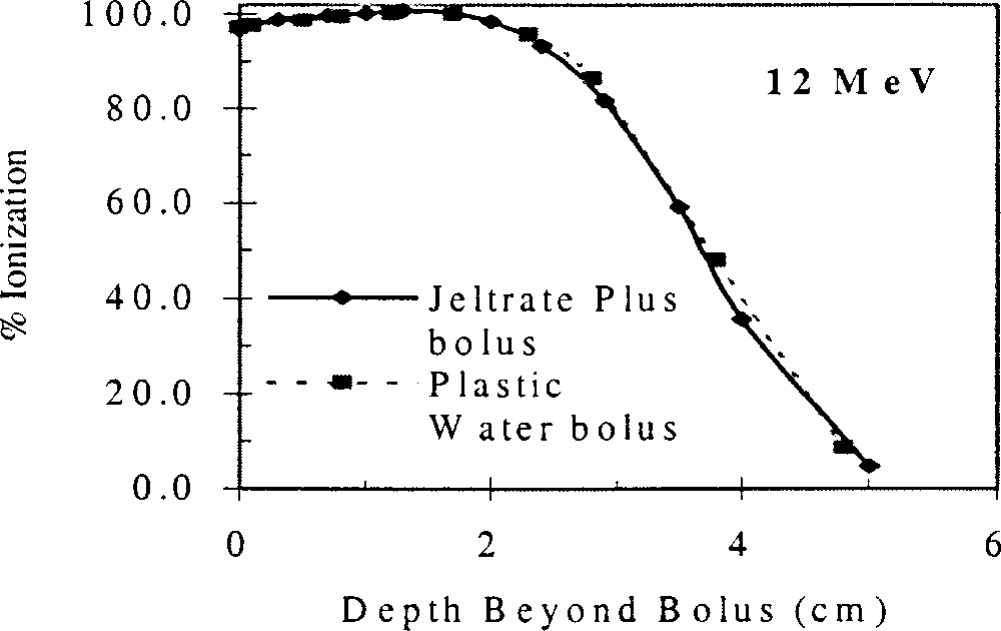
Central axis depth ionization curves for 12 MeV electrons measured beyond 1.3 cm of either Jeltrate®Plus bolus or Plastic Water® for a $10\times 10{\text{cm}}^{2}$ field and 100 cm SSD. The data for Jeltrate®Plus bolus are expressed as a percentage of the ionization at $d_{\max}$ in PlasticWater®.

**Figure 6:**
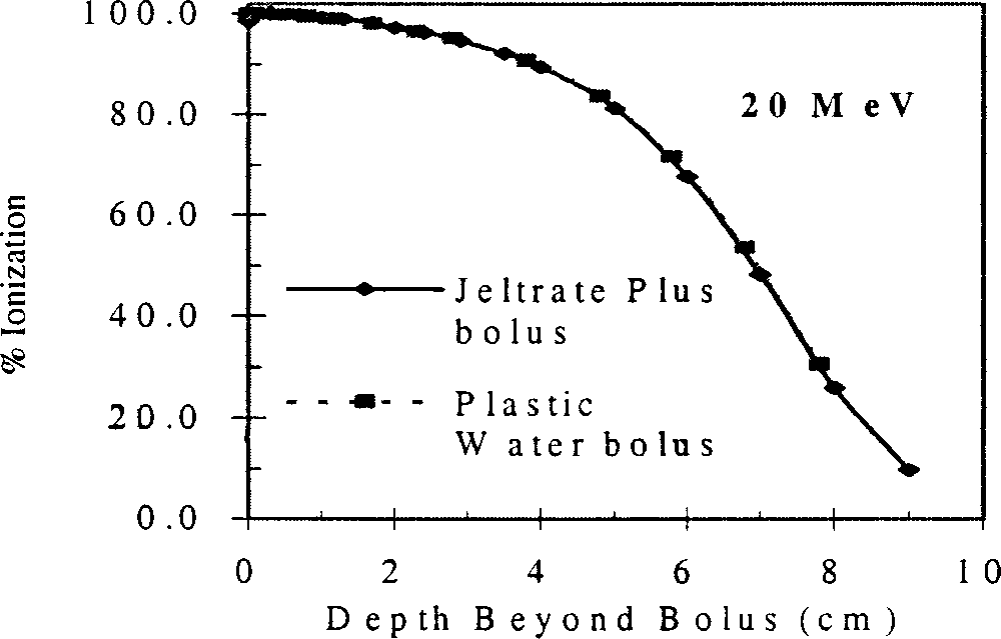
Central axis depth ionization curves for 20 MeV electrons measured beyond 1.3 cm of either Jeltrate®Plus bolus or Plastic Water® for a $10\times 10{\text{cm}}^{2}$ field and 100 cm SSD. The data for Jeltrate®Plus bolus are expressed as a percentage of the ionization at $d_{\max}$ in Plastic Water®.

Figures 4 through 6 show the results of the percent depth ionization measurements made in Plastic Water®, both with and without a 1.3 cm thick slab of Jeltrate®Plus placed on the surface. Figure 4 shows the results for 6 MeV electrons. Here the ionization reading at a certain depth under the 1.3 cm slab of Jeltrate®Plus bolus is related to the ionization reading at $d_{\max}$ in Plastic Water® and the resulting curve is compared directly with the percent depth ionization for Plastic Water®. To aid in the comparison of the depth ionization, the data for Plastic Water® are shifted by 1.3 cm. For 6 MeV electrons the depth ionization curve under bolus is within 1 mm of that for Plastic Water®. Similarly, for 12 MeV the depth difference in percent depth ionization data is 0.5 mm (see Fig. 5). For 20 MeV, the percent depth ionization data are identical under Jeltrate®Plus bolus or Plastic Water® (see Fig. 6). These results show that Jeltrate®Plus is an effective tissue equivalent bolus material, since the depth shifts of the percentage depth ionization curves are identical under an equal thickness (1.3 cm) of Jeltrate®Plus bolus or Plastic Water®.

The data in Table II show the results of measurements over a wider range of bolus thicknesses as might be encountered clinically to correct for missing tissue. The table presents the relative percent deviation of the measured ionization in Jeltrate®Plus versus Plastic Water® for 6, 12, and 20 MeV electron energies and 6 and 15 MV photon energies.

**Table II:** The percent deviation in ionization readings taken under various thicknesses of Jeltrate®Plus bolus relative to the readings under equal thicknesses of Plastic Water®. The data are for three electron and two photon beams.

Thickness (cm)	6 MeV	12 MeV	20 MeV	6 MV	15 MV
0.7	0.5	– 0.8	–0.4	–0.4	–0.2
1.3	0.0	–0.7	0.0	0.0	–0.2
2.0	–3.4	–0.3	0.0	–0.2	0.0
2.6	–6.7	0.0	0.2	–0.2	0.0
3.3		–1.0	0.3	–0.2	0.2
3.9		–2.5	0.5	0.0	0.2
4.6		–2.2	0.4	–0.2	0.2
5.2			0.5	0.0	0.0
5.9			0.0	–0.1	0.2
6.5			–0.8	–0.1	0.0
7.2			–2.8	0.3	0.0

It can be seen that the Jeltrate®Plus percent depth ionization data agree well with those measured in Plastic Water® for all photon and electron energies studied. For the electron beams, the deviation is consistently below 3.5% except for the measurement at a depth of 2.6 cm for 6 MeV electrons. The 6.7% difference at this depth can be attributed to the steep gradient of the percent depth ionization curve. This is of no major concern as the discrepancy occurs below a percent ionization of 25%, i.e., beyond the useful portion of the 6 MeV electron beam. For both photon beams, agreement in percent depth ionization data between Plastic Water® and Jeltrate®Plus is within 0.5%. The data in Table II also indicate that the Jeltrate®Plus bolus is more tissue equivalent than the polyether and hydrocolloid impression materials investigated previously.^1^

The observed similarity between the Jeltrate®Plus bolus and tissue is understandable when one considers additional characterization from computed tomography (CT) and elemental analysis. The 2:3 Jeltrate®Plus powder to water concentration has a physical density of $1020\;\text{kg}/m^{3}$ and a CT number of 44 Hounsfield Units (±21 over a $100{\text{mm}}^{2}$ region of interest measured on a GE LightSpeed Plus CT scanner, General Electric Medical, Milwaukee, WI). Soft tissue has CT numbers in the range of ∼40 to 60 HU, Plastic Water® in the range of 30 HU, and a mixture of Paraffin plus Bees wax (another bolus material used in our clinic) about 40 HU. Thus, the Jeltrate®Plus bolus has similar electron density to these materials.

The Jeltrate®Plus bolus with 2:3 powder to water concentration has an elemental composition of approximately 73% oxygen, 12% silicon, 7% hydrogen, 2% carbon and calcium (each), with trace amounts (<1%each) of potassium, sulphur, magnesium, sodium, phosphorous, titanium, and fluorine.^6^ The total stopping power of Jeltrate®Plus bolus (calculated using the NIST ESTAR database program)^7^ is less than 5% different than that of water for electron energies from 20 to 3 MeV (these energies span the range of the mean energy at depth between the surface and the depth of 80% ionization for the electron beams utilized).

In our clinic we have had good experience using Jeltrate®Plus as a bolus for irregular treatment areas such as the nose. At preparation, the room temperature mixture can be poured directly over the area of the patient that we need to bolus, and it sets in less than 2 min. There is no discomfort to the patient and a perfect impression is made. The bolus is then stored in a plastic container filled with water and once the patient is ready to begin treatment, a thin sheet of plastic Saran Wrap® is put over the treatment area and the bolus placed on top. In the past it was common practice to use Bee's wax and Paraffin wax to bolus an irregular treatment area. However, this was found to be very time consuming since an impression could not be made by pouring hot wax over the area of the patient that we wished to bolus. Instead the wax was poured into a mould and then carved with a knife to try and obtain the desired bolus shape.

## CONCLUSION

The Jeltrate®Plus bolus material is found to be stable over time both physically and dosimetrically when stored in a water filled container. Since the material is fairly inexpensive and can be quickly and easily made into different shapes for irregular treatment areas, it should be considered a viable alternative to common bolus materials such as Bee's wax and Paraffin wax. The optimal preparation method for a Jeltrate®Plus bolus has been determined to be a mixture of two parts powder to three parts water by weight. The physical density was found to be $1020\;\text{kg}/m^{3}$ and the CT number is 44±21 HU. Ionization readings under Jeltrate®Plus bolus made from 2:3 Jeltrate®Plus powder to water weight ratio, are identical to those under water equivalent plastic. That is, for both electron and photon beams, ionization readings are within 1% in ionization and 1 mm in depth over a range of thicknesses of clinical utility.

## References

[1] D. Dubois *et al*, “Moldable tissue equivalent bolus for high‐energy photon and electron therapy,” Med. Phys. 23 (9), 1547–1549 (1996). 8892252 10.1118/1.597820

[2] F. Khan , The Physics of Radiation Therapy, 2nd ed. (Williams and Wilkins, Baltimore, MD, 1984), p. 387.

[3] R. Moyer *et al*, “A Surface Bolus Material for High‐Energy Photon and Electron Therapy,” Radiology 146, 531–532 (1983). 6401364 10.1148/radiology.146.2.6401364

[4] Jeltrate®Plus Antimicrobial Alginate Impression Material (Dentsply Caulk, Milford, DE).

[5] Nuclear Associates Diagnostic Imaging and Radiation Therapy Catalog, Carle Place, NY, Nuclear Associates, 1998; pp. 315–318.

[6] Richard Bennett , Elemental analysis of dry Jeltrate ®Plus powder, Senior Process Associate, Dentsply Caulk, Milford, DE (private communication).

[7] M. J. Berger , J. S. Coursey , and M. A. Zucker , (1999). ESTAR, PSTAR, and ASTAR: Computer Programs for Calculating Stopping‐Power and Range Tables for Electrons, Protons, and Helium Ions (version 1.21), National Institute of Standards and Technology, Gaithersburg, MD, available at http://physics.nist.gov/Star (February 15, 2002).

